
JOURNAL INFORMATION
==============================
NLM Title Abbreviation: Behav Neurol
Journal ID: Behav Neurol
NLM Journal ID: 8914585
Journal ID: BN

Behavioural Neurology

ISSN: 0953-4180
EISSN: 1875-8584
Publisher: Wiley

ARTICLE INFORMATION
==============================
PMCID: PMC5215507
PMID: 22713401
DOI: 10.3233/BEN-2012-120258
Article ID: 376145
Article version: 1
Subjects: Other

New Insights from a Not-So-Neglected Field: Hemispatial Neglect

Hillis Argye E. 1 2 3 *
1 Departments of Neurology Johns Hopkins University School of Medicine Baltimore MD USA
2 Physical Medicine and Rehabilitation Johns Hopkins University School of Medicine Baltimore MD USA
3 Department of Cognitive Science Johns Hopkins University Baltimore MD USA
*Argye E. Hillis: argye@jhm1.edu
Print publication date: 2013
Volume: 26
Issue: 1-2
First page: 109
Last page: 110
Received 2012 Apr 23; Accepted 2012 Apr 23
Copyright: Copyright © 2013 Hindawi Publishing Corporation and the authors.
Copyright year: 2013
License: This is an open access article distributed under the Creative Commons Attribution License, which permits unrestricted use, distribution, and reproduction in any medium, provided the original work is properly cited.
License URL: https://creativecommons.org/licenses/by/3.0/

Funding: http://dx.doi.org/10.13039/100000065 National Institute of Neurological Disorders and Stroke R01 NS47691

==============================
Behavioural Neurology 26 (2013) 109–110
DOI 10.3233/BEN-2012-120258
IOS Press

### New insights from a not-so-neglected field: Hemispatial neglect

Argye E. Hillis[a,b,c,*]
[a]*Departments of Neurology, Johns Hopkins University School of Medicine, Baltimore, MD, USA*
[b]*Physical Medicine and Rehabilitation, Johns Hopkins University School of Medicine, Baltimore, MD, USA*
[c]*Department of Cognitive Science, Johns Hopkins University, Baltimore, MD, USA*

Hemispatial neglect remains one of the most remarkable syndromes investigated by behavioural neurologists. It occurs only after relatively focal (or at least asymmetric) brain damage, most commonly stroke, but is occasionally observed in asymmetric atrophy syndromes such as corticobasal syndrome or Posterior Cortical Atrophy due to Alzheimer's Disease pathology. It is also sometimes associated with other focal lesions such as tumor, abscess, head injury, or following resection. It is mild and transient in primates, even after large lesions, which reveals that only humans, who may have developed hemispheric asymmetry due to language, have hemispheric specialization for spatial attention or some aspect of attention such that a unilateral lesion causes marked hemispatial neglect. For example, Corbetta and Schulman [1] have proposed a model in which the dorsal spatial attention network is bilateral and symmetric, but the ventral nonspatial frontoparietal attention network is right-hemisphere dominant. Ventral lesions also cause physiological changes and disrupt connections to and within the spatial attention. Only a lesion to the ventral (right) network, which also affects the dorsal network, not the left dorsal network alone, causes marked hemispatial neglect. This model would account for more frequent and severe hemispatial neglect reported in many studies of neglect. However, Kleinman and colleagues [2] reported that hemispatial neglect is equally common after left and right hemisphere stroke, although the type of neglect observed is different after a lesion to the left versus the right hemisphere. Individuals with left hemisphere stroke were found to have stimulus-centered or object-centered neglect more often than viewer-centered neglect; while individuals with right hemisphere stroke were found to have the opposite: more frequent viewer-centered neglect. Viewer-centered neglect is easier to detect, as the person will neglect everything on the contralesional (usually left) side of his or her view, bump into things on the contralesional side of the body, fail to eat food on the contralesional side of the tray, and so on; while the person with stimulus-centered neglect will make more subtle errors in processing the contralesional side of individual stimuli on both sides of space (sometimes not recognized as hemispatial neglect). Therefore, the detection rate for viewer-centered neglect may be higher, accounting for the higher rate of neglect after right hemisphere stroke reported in some studies of neglect.

Severity of hemispatial neglect also increases with age, even after controlling for lesion volume [3], possibly due to worse ability to compensate with the intact hemisphere [4]. In fact, hemispatial neglect in children is rare, even after hemicraniotomy, including removal of the entire right cortex [5]. Severity of neglect also increases with the degree of leukoaraiosis, even after controlling for infarct size, consistent with the findings of the important role of lesions to white matter tracts in causing hemispatial neglect [6–9]. Thus, even though hemispatial neglect has been well-described and investigated for centuries, many new insights are being gained about the neural substrates underlying spatial attention and representation from investigating the various types of spatial neglect and related disorders and the factors that influence their occurrence and severity.

*Correspondence to: Argye E. Hillis, MD, Meyer 6-113, Johns Hopkins Hospital, Baltimore, MD 21287, USA. Tel.: +1 410 614 2381; Fax: +1 410 955 0672; E-mail: argye@JHMI.edu.

ISSN 0953-4180/13/$27.50 © 2013 – IOS Press and the authors. All rights reserved

Most of the literature on hemispatial neglect has focused on left visual neglect in near space (e.g. as tested on bedside examinations). However, visual neglect in far space, as well as tactile, auditory, motor, and olfactory neglect have been described [10,11]. In this issue, Aimiola and colleagues provide a novel investigation of near and far space. Heidler-Gary et al. report a double dissociation between left spatial motor extinction and left body motor extinction in individuals with acute stroke with somewhat distinct sites of lesions. Pitteri and colleagues demonstrate that tactile neglect can be distinguished from hemianesthesia with careful assessment.

Neglect frequently co-occurs with a variety of other cognitive deficits (such as anosognosia, impaired empathy, and aprosody), likely because the neural networks that support spatial attention or the cognitive processes that underlie spatial attention tasks used to test for hemispatial neglect overlap with those that support task switching, recognition of one's own deficits, recognition of facial expression and vocal intonation, theory of mind, and so on. Kleinman and colleagues report an investigation of perseveration versus hemispatial neglect, and the lesion sites associated with each in acute stroke. Invernizzi et al. describe a study of somatophrenia and anosognosia for hemiplegia. Finally, in a Clinical Note, Carota et al. report a patient with anosognosia, attention blindsight, and left neglect. The special issue also includes an important paper by Ishizaki and co-workers of impaired visual-spatial attention in Alzheimer's Disease, which shows how a symmetric neurodegenerative disease results in impaired shifting of visual spatial attention, but not hemispatial neglect. Together, these novel studies of neglect and related disorders provide new insights into brain-behavior relationships on the basis of detailed analysis of patient performance (and in many cases, their lesion sites).

### Acknowledgments

The author and the some of the work reported herein were supported by NIH/NINDS R01 NS47691.

### References

[1] M. Corbetta and C.L. Shulman, Spatial neglect and attention networks, *Annu Rev Neurosci* **34** (2011), 569–599.

[2] J.T. Kleinman, M. Newhart, C. Davis, J. Heidler-Gary, R. Gottesman and A.E. Hillis, Right hemispatial neglect: frequency and characterization following acute left hemisphere stroke, *Brain Cogn* **64** (2007), 50–59.

[3] R.F. Gottesman, J.T. Kleinman, C. Davis, J. Heidler-Gary, M. Newhart, V. Kannan and A.E. Hillis, Unilateral neglect is more severe and common in older patients with right-hemispheric stroke, *Neurology* **71** (2008), 1439–1444.

[4] D.N. Levine, J.D. Warach, L. Benowitz and R. Calvanio, Left spatial neglect: effects of lesion size and premorbid brain atrophy on severity and recovery following right cerebral infarction, *Neurology* **36**(3) (1986 Mar), 362–366.

[5] E.B. Marsh, M. Newhart, J.T. Kleinman, J. Heidler-Gary, E.P. Vining, J.M. Freeman, E.H. Kossoff and A.E. Hillis, Hemispherectomy sustained before adulthood does not cause persistent hemispatial neglect, *Cortex* **45**(5) (2009), 677–685.

[6] N. Shinoura, Y. Suzuki, R. Yamada, Y. Tabei, K. Saito and K. Yagi, Damage to the right superior longitudinal fasciculus in the inferior parietal lobe plays a role in spatial neglect, *Neuropsychologia* **47** (2009), 2600–2603.

[7] F. Doricchi and F. Tomaiuolo, The anatomy of neglect without hemianopia: a key role for parietal-frontal disconnection? *Neuroreport* **14** (2003), 2239–2243.

[8] M. Thiebaut de Schotten, M. Urbanski et al., Direct evidence for a parietal-frontal pathway subserving spatial awareness in humans, *Science* **309** (2005), 2226–2228.

[9] P. Bartolomeo, M. Thiebaut de Schotten and F. Doricchi, Left unilateral neglect as a disconnection syndrome, *Cerebral Cortex* **17** (2007), 2479–2490.

[10] A.E. Hillis, F.A. Lenz, T.A. Zirh, P.M. Dougherty, T.S. Eckel and K. Jackson, Hemispatial somatosensory and motor extinction after stereotactic thalamic lesions, *Neurocase* **4** (1998), 21–34.

[11] E. Valenstein and K.M. Heilman, Unilateral hypokinesia and motor extinction, *Neurology* **31** (1981), 445–448.

[12] A. Carota, F. Bianchini, P. Calabrese and L. Pizzamiglio, The "Altitudinal Anton's syndrome": coexistence of anosognosia, attention blindsight and left spatial neglect, *Behavioural Neurology* **26** (2013), 157–163.

[13] Z. Bahrainwala, A.E. Hillis, J. Dearborn and R.F. Gottesman, Effect of leukoaraiosis on neglect performance in acute stroke patients, Submitted.

[14] J.T. Kleinman, J.C. DuBois, M. Newhart, R.A. Popat and A.E. Hillis, Disentangling the Neuroanatomical Correlates of Perseveration from Unilateral Spatial Neglect, *Behavioural Neurology* **26** (2013), 131–138.

[15] J. Ishizaki, K. Meguro, N. Nara, M. Kasai and A. Yamadori, Impaired shifting of visuospatial attention in Alzheimer's disease as shown by the covert orienting paradigm: Implications for visual construction disability, *Behavioural Neurology* **26** (2013), 121–129.

[16] L. Aimola, I. Schindler and A. Venneri, Task- and response related dissociations between neglect in near and far space: A morphometric case study, *Behavioural Neurology*, in press.

[17] M. Pitteri, A. Venneri, F. Meneghello and K. Priftis, How to differentiate hemianesthesia from left tactile neglect: A preliminary case report. In press, *Behavioural Neurology* **26** (2013), 151–155.

[18] P. Invernizzi, M. Gandola, D. Romano, L. Zapparoli, G. Bottini and E. Paulesu, What is mine? Behavioral and anatomical dissociations between somatoparaphrenia and anosognosia for hemiplegia, *Behavioural Neurology* **26** (2013), 139–150.

[19] J. Heidler-Gary, M. Pawlak, E.H. Herskovits, M. Newhart, C. Davis, L.A. Trupe and A.E. Hillis, Motor Extinction in Distinct Reference Frames: A Double Dissociation, *Behavioural Neurology* **26** (2013), 111–119.
